# Supplementary material for: Intimate partner violence: A key correlate of women’s physical and mental health in informal settlements in Nairobi, Kenya
Source: PLoS One. 2020 Apr 2;15(4):e0230894. doi: 10.1371/journal.pone.0230894 (PMC7117691; doi:10.1371/journal.pone.0230894)
Supplement: S1 Appendix — (DOCX) [file pone.0230894.s001.docx]

**S1 Appedix**

**Table A – Results from logistic regression of women’s mental health on sociodemographic characterstics and IPV (n=361)**

|  | Binary Mental Health Score | | | | | | Binary Psychosocial Distress | | | | | |
| --- | --- | --- | --- | --- | --- | --- | --- | --- | --- | --- | --- | --- |
|  | Model 1 | | | Model 2 | | | Model 1 | | | Model 2 | | |
|  | Adj-OR | p-value | CI(95%) | Adj-OR | p-value | CI(95%) | OR | p-value | CI(95%) | OR | p-value | CI(95%) |
| Socio-economic variables |  |  |  |  |  |  |  |  |  |  |  |  |
| Monthly household income |  |  |  |  |  |  |  |  |  |  |  |  |
| 10,000 – 15,000 KES/month | 0.81 | 0.427 | 0.477-1.368 | 0.7 | 0.196 | 0.402-1.206 | 0.57 | 0.037 | 0.339-0.968 | 0.71 | 0.228 | 0.406-1.241 |
| More than 15,000 KES/month | 0.9 | 0.746 | 0.467-1.727 | 0.75 | 0.397 | 0.384-1.463 | 0.41 | 0.006 | 0.216-0.775 | 0.55 | 0.072 | 0.29-1.054 |
| Aware of household finances | 0.72 | 0.477 | 0.284-1.802 | 0.6 | 0.278 | 0.234-1.519 | 1.2 | 0.694 | 0.477-3.034 | 1.74 | 0.246 | 0.683-4.418 |
| Education |  |  |  |  |  |  |  |  |  |  |  |  |
| Completed primary school | 1.13 | 0.694 | 0.62-2.052 | 1.08 | 0.796 | 0.587-2.003 | 1.06 | 0.834 | 0.594-1.908 | 1.16 | 0.616 | 0.642-2.111 |
| At least some secondary | 0.93 | 0.783 | 0.536-1.6 | 0.9 | 0.719 | 0.521-1.57 | 1.02 | 0.935 | 0.599-1.745 | 1.03 | 0.927 | 0.59-1.785 |
| Marital status |  |  |  |  |  |  |  |  |  |  |  |  |
| Living with a man, not married | 0.8 | 0.595 | 0.346-1.839 | 0.74 | 0.5 | 0.305-1.786 | 1.11 | 0.816 | 0.464-2.653 | 1.21 | 0.692 | 0.469-3.125 |
| Regular partner, live apart | 0.95 | 0.865 | 0.501-1.787 | 0.78 | 0.451 | 0.401-1.502 | 0.76 | 0.4 | 0.399-1.443 | 1.03 | 0.925 | 0.522-2.043 |
| Casual boyfriend | 0.26 | 0.017 | 0.083-0.783 | 0.23 | 0.016 | 0.069-0.759 | 1.13 | 0.813 | 0.408-3.128 | 1.3 | 0.634 | 0.44-3.848 |
| Number of children |  |  |  |  |  |  |  |  |  |  |  |  |
| 1-2 children | 0.72 | 0.395 | 0.338-1.535 | 0.82 | 0.608 | 0.374-1.78 | 1.54 | 0.245 | 0.745-3.166 | 1.14 | 0.728 | 0.539-2.422 |
| 3-4 children | 0.34 | 0.012 | 0.144-0.783 | 0.37 | 0.022 | 0.154-0.866 | 1.38 | 0.441 | 0.61-3.105 | 1.12 | 0.786 | 0.479-2.639 |
| 5 or more children | 0.24 | 0.011 | 0.08-0.716 | 0.31 | 0.041 | 0.1-0.951 | 1.84 | 0.259 | 0.638-5.295 | 1.23 | 0.71 | 0.41-3.703 |
| Age | 1.01 | 0.438 | 0.981-1.044 | 1.01 | 0.643 | 0.976-1.041 | 0.99 | 0.569 | 0.959-1.023 | 1 | 0.851 | 0.963-1.031 |
| Respondent is employed | 1.19 | 0.472 | 0.736-1.94 | 1.24 | 0.388 | 0.759-2.03 | 1.18 | 0.493 | 0.736-1.889 | 1.02 | 0.924 | 0.622-1.688 |
| Respondent has a business | 0.65 | 0.101 | 0.389-1.088 | 0.68 | 0.156 | 0.399-1.159 | 1.73 | 0.037 | 1.034-2.902 | 1.44 | 0.194 | 0.829-2.513 |
| Access to toilet |  |  |  |  |  |  |  |  |  |  |  |  |
| Has access to a toilet at all times | 1.91 | 0.011 | 1.164-3.139 | 1.77 | 0.027 | 1.067-2.942 | 0.47 | 0.002 | 0.288-0.751 | 0.52 | 0.013 | 0.316-0.871 |
| Access to water |  |  |  |  |  |  |  |  |  |  |  |  |
| outside tap/well | 2.93 | 0.033 | 1.09-7.881 | 3.17 | 0.022 | 1.18-8.507 | 0.62 | 0.298 | 0.247-1.535 | 0.56 | 0.262 | 0.206-1.538 |
| public tap/well | 3.08 | 0.025 | 1.151-8.215 | 3.46 | 0.013 | 1.306-9.189 | 0.52 | 0.15 | 0.212-1.27 | 0.41 | 0.084 | 0.153-1.125 |
| vendor/tanker/burst pipe | 2.85 | 0.117 | 0.768-10.564 | 2.87 | 0.122 | 0.753-10.915 | 0.26 | 0.031 | 0.078-0.883 | 0.25 | 0.04 | 0.066-0.939 |
| Violence Variables |  |  |  |  |  |  |  |  |  |  |  |  |
| Intimate partner psychological violence |  |  |  | 0.81 | 0.441 | 0.469-1.391 |  |  |  | 1.53 | 0.13 | 0.882-2.656 |
| Intimate partner sexual violence |  |  |  | 0.82 | 0.488 | 0.467-1.438 |  |  |  | 2.39 | 0.004 | 1.328-4.294 |
| Intimate partner physical violence |  |  |  | 0.68 | 0.151 | 0.397-1.153 |  |  |  | 1.05 | 0.871 | 0.595-1.844 |
| Model Comparison Statistics |  |  |  |  |  |  |  |  |  |  |  |  |
| Wald test statistic, F(p-value) |  |  | 2.26(p = .003) |  |  | 2.00(p = .114) |  |  | 2.06(p = 0.007) |  |  | 7.15(p = 0.000) |

|  | Major Depressive Disorder (MDD) | | | | | | Suicidality | | | | | |
| --- | --- | --- | --- | --- | --- | --- | --- | --- | --- | --- | --- | --- |
|  | Model 1 | | | Model 2 | | | Model 1 | | | Model 2 | | |
|  | OR | p-value | CI(95%) | OR | p-value | CI(95%) | OR | p-value | CI(95%) | OR | p-value | CI(95%) |
| Socio-economic variables |  |  |  |  |  |  |  |  |  |  |  |  |
| Monthly household income |  |  |  |  |  |  |  |  |  |  |  |  |
| 10,000 – 15,000 KES/month | 0.77 | 0.435 | 0.397-1.488 | 1.22 | 0.575 | 0.607-2.456 | 0.74 | 0.318 | 0.411-1.335 | 1.07 | 0.846 | 0.563-2.017 |
| More than 15,000 KES/month | 0.19 | 0.007 | 0.06-0.632 | 0.35 | 0.088 | 0.105-1.17 | 0.62 | 0.228 | 0.282-1.352 | 1.02 | 0.969 | 0.48-2.147 |
| Aware of household finances | 0.59 | 0.363 | 0.194-1.824 | 0.97 | 0.968 | 0.267-3.56 | 0.76 | 0.565 | 0.29-1.966 | 1.13 | 0.822 | 0.403-3.143 |
| Education |  |  |  |  |  |  |  |  |  |  |  |  |
| Completed primary school | 1 | 0.991 | 0.456-2.174 | 1.19 | 0.687 | 0.507-2.798 | 1.43 | 0.289 | 0.736-2.792 | 1.84 | 0.111 | 0.87-3.873 |
| At least some secondary | 0.54 | 0.09 | 0.263-1.101 | 0.54 | 0.113 | 0.256-1.155 | 0.91 | 0.774 | 0.487-1.709 | 1.02 | 0.951 | 0.511-2.046 |
| Marital status |  |  |  |  |  |  |  |  |  |  |  |  |
| Living with a man, not married | 0.45 | 0.202 | 0.129-1.544 | 0.48 | 0.301 | 0.121-1.923 | 0.56 | 0.278 | 0.194-1.604 | 0.59 | 0.391 | 0.172-1.99 |
| Regular partner, live apart | 0.66 | 0.369 | 0.261-1.647 | 1.3 | 0.579 | 0.514-3.287 | 0.85 | 0.683 | 0.39-1.854 | 1.56 | 0.301 | 0.669-3.66 |
| Casual boyfriend | 1.63 | 0.403 | 0.518-5.127 | 2.14 | 0.253 | 0.58-7.894 | 1.43 | 0.53 | 0.469-4.349 | 1.84 | 0.327 | 0.544-6.209 |
| Number of children |  |  |  |  |  |  |  |  |  |  |  |  |
| 1-2 children | 0.93 | 0.88 | 0.336-2.551 | 0.63 | 0.406 | 0.207-1.894 | 0.74 | 0.495 | 0.317-1.743 | 0.51 | 0.166 | 0.197-1.324 |
| 3-4 children | 1.04 | 0.942 | 0.332-3.276 | 0.75 | 0.664 | 0.208-2.721 | 1.26 | 0.636 | 0.486-3.253 | 1.01 | 0.987 | 0.353-2.88 |
| 5 or more children | 1.61 | 0.502 | 0.401-6.454 | 0.81 | 0.804 | 0.158-4.18 | 1.66 | 0.392 | 0.521-5.271 | 0.81 | 0.753 | 0.212-3.07 |
| Age | 0.99 | 0.58 | 0.947-1.031 | 1.01 | 0.815 | 0.959-1.055 | 1 | 0.946 | 0.964-1.035 | 1.02 | 0.41 | 0.978-1.056 |
| Respondent is employed | 1.15 | 0.683 | 0.589-2.243 | 1.18 | 0.685 | 0.53-2.625 | 0.89 | 0.682 | 0.503-1.568 | 0.88 | 0.711 | 0.457-1.707 |
| Respondent has a business | 2.96 | 0.002 | 1.514-5.785 | 2.84 | 0.005 | 1.381-5.852 | 1.18 | 0.597 | 0.64-2.17 | 1.1 | 0.765 | 0.577-2.111 |
| Access to toilet |  |  |  |  |  |  |  |  |  |  |  |  |
| Has access to a toilet at all times | 0.77 | 0.439 | 0.399-1.491 | 1.21 | 0.592 | 0.599-2.452 | 0.74 | 0.271 | 0.431-1.268 | 0.97 | 0.931 | 0.536-1.771 |
| Access to water |  |  |  |  |  |  |  |  |  |  |  |  |
| outside tap/well | 1.5 | 0.479 | 0.487-4.616 | 1 | 0.997 | 0.243-4.128 | 0.78 | 0.629 | 0.285-2.136 | 0.49 | 0.267 | 0.139-1.727 |
| public tap/well | 0.52 | 0.271 | 0.158-1.681 | 0.34 | 0.16 | 0.076-1.533 | 0.52 | 0.206 | 0.188-1.433 | 0.31 | 0.072 | 0.085-1.113 |
| vendor/tanker/burst pipe | 2.53 | 0.176 | 0.658-9.755 | 2.62 | 0.281 | 0.454-15.093 | 1.81 | 0.351 | 0.519-6.307 | 1.67 | 0.502 | 0.372-7.507 |
| Violence Variables |  |  |  |  |  |  |  |  |  |  |  |  |
| Intimate partner psychological violence |  |  |  | 2.63 | 0.011 | 1.247-5.543 |  |  |  | 2.36 | 0.01 | 1.224-4.535 |
| Intimate partner sexual violence |  |  |  | 1.51 | 0.308 | 0.683-3.34 |  |  |  | 1.27 | 0.506 | 0.624-2.597 |
| Intimate partner physical violence |  |  |  | 3.14 | 0.002 | 1.504-6.569 |  |  |  | 3.74 | 0 | 1.833-7.622 |
| Model Comparison Statistics |  |  |  |  |  |  |  |  |  |  |  |  |
| Wald test statistic, F(p-value) |  |  | 2.20(p = .004) |  |  | 10.56(p = .000) |  |  | 1.16(p = .292) |  |  | 11.95(p = .000) |

|  | Alcohol Use | | | | | | Tobacco Use | | | | | |
| --- | --- | --- | --- | --- | --- | --- | --- | --- | --- | --- | --- | --- |
|  | Model 1 | | | Model 2 | | | Model 1 | | | Model 2 | | |
|  | OR | p-value | CI(95%) | OR | p-value | CI(95%) | OR | p-value | CI(95%) | OR | p-value | CI(95%) |
| Socio-economic variables |  |  |  |  |  |  |  |  |  |  |  |  |
| Monthly household income |  |  |  |  |  |  |  |  |  |  |  |  |
| 10,000 – 15,000 KES/month | 2.09 | 0.03 | 1.073-4.089 | 2.21 | 0.019 | 1.139-4.299 | 6.1 | 0.003 | 1.876-19.839 | 8.8 | 0.001 | 2.353-32.896 |
| More than 15,000 KES/month | 3.39 | 0.003 | 1.497-7.692 | 3.86 | 0.001 | 1.685-8.827 | 2.48 | 0.263 | 0.504-12.153 | 3.75 | 0.092 | 0.807-17.388 |
| Aware of household finances | 4.07 | 0.115 | 0.71-23.261 | 5.6 | 0.094 | 0.746-42.008 | 3.82 | 0.442 | 0.125-117.084 | 5.04 | 0.45 | 0.075-337.026 |
| Education |  |  |  |  |  |  |  |  |  |  |  |  |
| Completed primary school | 0.7 | 0.354 | 0.322-1.501 | 0.83 | 0.655 | 0.373-1.86 | 0.97 | 0.958 | 0.34-2.784 | 1.57 | 0.456 | 0.481-5.097 |
| At least some secondary | 1.37 | 0.35 | 0.71-2.628 | 1.47 | 0.262 | 0.749-2.885 | 1.07 | 0.896 | 0.369-3.127 | 1.15 | 0.808 | 0.371-3.563 |
| Marital status |  |  |  |  |  |  |  |  |  |  |  |  |
| Living with a man, not married | 0.71 | 0.582 | 0.204-2.447 | 0.76 | 0.648 | 0.23-2.498 | 2.69 | 0.19 | 0.611-11.823 | 3.58 | 0.122 | 0.711-18.072 |
| Regular partner, live apart | 1.75 | 0.167 | 0.791-3.88 | 2.25 | 0.048 | 1.008-5.012 | 1.23 | 0.806 | 0.232-6.556 | 1.96 | 0.42 | 0.381-10.109 |
| Casual boyfriend | 3.05 | 0.043 | 1.034-8.986 | 3.21 | 0.049 | 1.004-10.257 | 2.79 | 0.325 | 0.361-21.556 | 3.08 | 0.331 | 0.318-29.851 |
| Number of children |  |  |  |  |  |  |  |  |  |  |  |  |
| 1-2 children | 2.32 | 0.142 | 0.755-7.151 | 2.26 | 0.174 | 0.697-7.319 | 1.12 | 0.908 | 0.168-7.437 | 1.07 | 0.946 | 0.145-7.911 |
| 3-4 children | 2.05 | 0.256 | 0.593-7.095 | 2.17 | 0.235 | 0.603-7.847 | 1.26 | 0.827 | 0.153-10.486 | 1.36 | 0.787 | 0.149-12.375 |
| 5 or more children | 2.13 | 0.333 | 0.46-9.857 | 1.88 | 0.447 | 0.369-9.567 | 3.61 | 0.274 | 0.361-35.991 | 3.96 | 0.258 | 0.363-43.185 |
| Age | 1 | 0.928 | 0.961-1.044 | 1.01 | 0.753 | 0.963-1.053 | 0.94 | 0.261 | 0.845-1.047 | 0.94 | 0.315 | 0.84-1.058 |
| Respondent is employed | 1.16 | 0.627 | 0.633-2.135 | 1.12 | 0.727 | 0.596-2.098 | 2.14 | 0.101 | 0.861-5.33 | 2.04 | 0.091 | 0.892-4.663 |
| Respondent has a business | 0.76 | 0.395 | 0.405-1.43 | 0.67 | 0.256 | 0.339-1.334 | 4.22 | 0.006 | 1.525-11.658 | 3.82 | 0.012 | 1.34-10.906 |
| Access to toilet |  |  |  |  |  |  |  |  |  |  |  |  |
| Has access to a toilet at all times | 0.42 | 0.012 | 0.213-0.828 | 0.52 | 0.06 | 0.265-1.028 | 0.76 | 0.579 | 0.289-2.001 | 1.05 | 0.935 | 0.361-3.029 |
| Access to water |  |  |  |  |  |  |  |  |  |  |  |  |
| outside tap/well | 0.31 | 0.059 | 0.092-1.044 | 0.3 | 0.066 | 0.084-1.084 | 0.63 | 0.575 | 0.127-3.15 | 0.59 | 0.557 | 0.101-3.45 |
| public tap/well | 0.71 | 0.574 | 0.221-2.307 | 0.69 | 0.534 | 0.208-2.258 | 0.68 | 0.621 | 0.144-3.189 | 0.5 | 0.368 | 0.111-2.261 |
| vendor/tanker/burst pipe | 1.91 | 0.403 | 0.419-8.672 | 2.32 | 0.319 | 0.443-12.118 | 2.99 | 0.294 | 0.385-23.23 | 3.03 | 0.347 | 0.299-30.757 |
| Violence Variables |  |  |  |  |  |  |  |  |  |  |  |  |
| Intimate partner psychological violence |  |  |  | 2.63 | 0.006 | 1.315-5.24 |  |  |  | 3.77 | 0.025 | 1.179-12.049 |
| Intimate partner sexual violence |  |  |  | 1.07 | 0.843 | 0.541-2.12 |  |  |  | 1.33 | 0.607 | 0.448-3.958 |
| Intimate partner physical violence |  |  |  | 0.82 | 0.546 | 0.434-1.556 |  |  |  | 0.8 | 0.665 | 0.282-2.246 |
| Model Comparison Statistics |  |  |  |  |  |  |  |  |  |  |  |  |
| Wald test statistic, F(p-value) |  |  | 1.88(p = .017) |  |  | 2.73(p = .044) | 2.02 |  | 1.90(p = .015) |  |  | 2.06(p = .105) |

**Table B – Results from logistic regression of women’s physical health on sociodemographic characterstics and IPV (n=361)**

|  | Binary Physical Health Score | | | | | | Reproductive Health | | | | | |
| --- | --- | --- | --- | --- | --- | --- | --- | --- | --- | --- | --- | --- |
|  | Model 1 | | | Model 2 | | | Model 1 | | | Model 2 | | |
|  | Adj-OR | p-value | CI(95%) | Adj-OR | p-value | CI(95%) | Adj-OR | p-value | CI(95%) | Adj-OR | p-value | CI(95%) |
| Socio-economic variables |  |  |  |  |  |  |  |  |  |  |  |  |
| Monthly household income |  |  |  |  |  |  |  |  |  |  |  |  |
| 10,000 – 15,000 KES/month | 0.9 | 0.689 | 0.534-1.514 | 0.75 | 0.291 | 0.435-1.284 | 0.7 | 0.168 | 0.415-1.166 | 0.82 | 0.462 | 0.481-1.395 |
| More than 15,000 KES/month | 1.57 | 0.165 | 0.831-2.965 | 1.32 | 0.421 | 0.672-2.592 | 0.6 | 0.101 | 0.326-1.106 | 0.74 | 0.353 | 0.391-1.399 |
| Aware of household finances | 2.71 | 0.048 | 1.007-7.315 | 2.44 | 0.07 | 0.928-6.434 | 1.08 | 0.868 | 0.437-2.664 | 1.38 | 0.491 | 0.548-3.496 |
| Education |  |  |  |  |  |  |  |  |  |  |  |  |
| Completed primary school | 1.18 | 0.584 | 0.65-2.147 | 1.24 | 0.499 | 0.662-2.331 | 1.3 | 0.356 | 0.747-2.251 | 1.35 | 0.296 | 0.766-2.396 |
| At least some secondary | 0.93 | 0.781 | 0.545-1.579 | 0.92 | 0.773 | 0.533-1.597 | 0.69 | 0.165 | 0.404-1.168 | 0.67 | 0.15 | 0.389-1.156 |
| Marital status |  |  |  |  |  |  |  |  |  |  |  |  |
| Living with a man, not married | 0.72 | 0.463 | 0.3-1.729 | 0.63 | 0.345 | 0.238-1.653 | 0.76 | 0.492 | 0.343-1.675 | 0.78 | 0.563 | 0.342-1.794 |
| Regular partner, live apart | 0.93 | 0.829 | 0.489-1.776 | 0.78 | 0.467 | 0.404-1.517 | 1.05 | 0.879 | 0.579-1.893 | 1.28 | 0.445 | 0.681-2.394 |
| Casual boyfriend | 0.55 | 0.226 | 0.213-1.441 | 0.5 | 0.157 | 0.193-1.304 | 0.94 | 0.906 | 0.324-2.717 | 1.01 | 0.984 | 0.353-2.894 |
| Number of children |  |  |  |  |  |  |  |  |  |  |  |  |
| 1-2 children | 0.75 | 0.458 | 0.357-1.591 | 0.79 | 0.559 | 0.355-1.752 | 1.31 | 0.449 | 0.649-2.655 | 1.08 | 0.823 | 0.534-2.201 |
| 3-4 children | 0.48 | 0.086 | 0.205-1.112 | 0.5 | 0.124 | 0.203-1.214 | 0.72 | 0.438 | 0.318-1.644 | 0.62 | 0.257 | 0.272-1.417 |
| 5 or more children | 0.53 | 0.236 | 0.189-1.509 | 0.65 | 0.452 | 0.211-2.003 | 0.83 | 0.717 | 0.314-2.219 | 0.65 | 0.396 | 0.245-1.744 |
| Age | 0.98 | 0.205 | 0.955-1.01 | 0.98 | 0.138 | 0.949-1.007 | 0.99 | 0.479 | 0.963-1.018 | 0.99 | 0.663 | 0.965-1.023 |
| Respondent is employed | 0.97 | 0.908 | 0.607-1.559 | 0.99 | 0.974 | 0.605-1.625 | 1.06 | 0.797 | 0.659-1.721 | 0.95 | 0.843 | 0.576-1.569 |
| Respondent has a business | 0.6 | 0.049 | 0.357-0.998 | 0.57 | 0.04 | 0.333-0.975 | 1.36 | 0.239 | 0.814-2.284 | 1.18 | 0.554 | 0.687-2.013 |
| Access to toilet |  |  |  |  |  |  |  |  |  |  |  |  |
| Has access to a toilet at all times | 1.31 | 0.263 | 0.817-2.098 | 1.38 | 0.205 | 0.84-2.257 | 0.79 | 0.323 | 0.49-1.266 | 0.87 | 0.578 | 0.529-1.426 |
| Access to water |  |  |  |  |  |  |  |  |  |  |  |  |
| outside tap/well | 1.53 | 0.372 | 0.601-3.888 | 1.93 | 0.187 | 0.725-5.138 | 1.31 | 0.592 | 0.486-3.54 | 1.3 | 0.593 | 0.496-3.408 |
| public tap/well | 1.65 | 0.289 | 0.652-4.203 | 2.26 | 0.103 | 0.848-6.006 | 1.85 | 0.214 | 0.7-4.912 | 1.69 | 0.274 | 0.659-4.354 |
| vendor/tanker/burst pipe | 1.63 | 0.432 | 0.482-5.49 | 2.07 | 0.256 | 0.589-7.287 | 1.69 | 0.408 | 0.489-5.807 | 1.79 | 0.341 | 0.538-5.982 |
| Violence Variables |  |  |  |  |  |  |  |  |  |  |  |  |
| Intimate partner psychological violence |  |  |  | 1.7 | 0.063 | 0.972-2.956 |  |  |  | 1.23 | 0.444 | 0.723-2.09 |
| Intimate partner sexual violence |  |  |  | 0.83 | 0.504 | 0.473-1.446 |  |  |  | 1.97 | 0.022 | 1.105-3.519 |
| Intimate partner physical violence |  |  |  | 0.36 | 0 | 0.21-0.613 |  |  |  | 0.95 | 0.862 | 0.554-1.639 |
| Model Comparison Statistics |  |  |  |  |  |  |  |  |  |  |  |  |
| Wald test statistic, F(p-value) |  |  | 1.42(p = .121) |  |  | 5.87(p = .001) |  |  | 1.00(p = .458) |  |  | 3.28(p = .021) |
