# Supplementary material for: Intimate partner violence: A key correlate of women’s physical and mental health in informal settlements in Nairobi, Kenya
Source: PLoS One. 2020 Apr 2;15(4):e0230894. doi: 10.1371/journal.pone.0230894 (PMC7117691; doi:10.1371/journal.pone.0230894)
Supplement: Attachment — Submitted filename: Response to Reviewers.docx [file pone.0230894.s004.docx]

**Response to Reviewers’ Comments**

We want to thank the reviewer, again, for the insightful recommendations. We have made the appropriate adjustments to the paper and responded to the specific comments below. ***Please note:*** line numbers referenced here refer to the *clean* document (with all track-changes accepted).

**Reviewer #1**

*Comment 1:*
Line 83- “developing countries” (which I admit I used in my own review of this paper) may not be the most appropriate term. Check with PLOS for their preferred term to refer to what are commonly referred to “low- and middle-income countries” or LMIC.

*Response:*

Thank you for this comment. We have made the adjustment to use LMIC. (See ln 83)

*Comment 2:*
Line 138- the same sentence regarding the Gibbs article from earlier in the introduction is mentioned again here- one reference is likely sufficient

*Response:*

We have made the adjustment.

*Comment 3:*
Line 234: a comma should be placed after “study”

*Response:*

We have make the adjustment.

*Comment 4:*
I am concerned that substantial information is being lost in the SF-36 by dichotomizing at the median. Is there a reason the authors decided to dichotomize instead of leaving it as a continuous variable and using linear regression for these models? A sentence expounding on this decision prior to its justification using prior studies would be helpful.

*Response:*

We really appreciate this point and spent a great deal of time considering our approach to this issue both in the first revision and in this one. While some information may be lost in the dichotomization of the SF-36 at the median, the combined physical health and mental health components are not normally distributed and cannot be transformed in such a way to ensure the normality assumptions of a linear regression are being met; thus, the findings in the linear regression models may be biased or not interpretable. Following previous methods employing the SF-36, we felt the best approach to ensure the results were meaningful and accurate was to dichotomize the scores [1-3]. However, we have also acknowledged this limitation in the limitations section (please see lines 428-431).

**References**

1.	Casso D, Buist DS, Taplin S. Quality of life of 5–10 year breast cancer survivors diagnosed between age 40 and 49. Health and quality of life outcomes. 2004;2(1):25.

2.	Cioncoloni D, Innocenti I, Bartalini S, Santarnecchi E, Rossi S, Rossi A, et al. Individual factors enhance poor health-related quality of life outcome in multiple sclerosis patients. Significance of predictive determinants. Journal of the neurological sciences. 2014;345(1-2):213-9.

3.	Sineke T, Evans D, Schnippel K, van Aswegen H, Berhanu R, Musakwa N, et al. The impact of adverse events on health-related quality of life among patients receiving treatment for drug-resistant tuberculosis in Johannesburg, South Africa. Health and quality of life outcomes. 2019;17(1):94. doi: 10.1186/s12955-019-1155-4.
